# Microbiome and Metabolome Analyses Reveal the Disruption of Lipid Metabolism in Systemic Lupus Erythematosus

**DOI:** 10.3389/fimmu.2020.01703

**Published:** 2020-07-31

**Authors:** Jingquan He, Tianlong Chan, Xiaoping Hong, Fengping Zheng, Chengxin Zhu, Lianghong Yin, Weier Dai, Donge Tang, Dongzhou Liu, Yong Dai

**Affiliations:** ^1^Department of Clinical Medical Research Center, The Second Clinical Medical College of Jinan University (Shenzhen People's Hospital), Shenzhen, China; ^2^Biotree Institute of Health, Biotree, Shanghai, China; ^3^Department of Nephrology, The First Affiliated Hospital of Jinan University, Guangzhou, China; ^4^College of Natural Science, University of Texas at Austin, Austin, TX, United States

**Keywords:** systemic lupus erythematosus, gut microbiome, metabolome, lipid metabolism, bile acids

## Abstract

Systemic lupus erythematosus (SLE) is a systemic autoimmune disease that affects thousands of people worldwide. Recently, alterations in metabolism and gut microbiome have emerged as key regulators of SLE pathogenesis. However, it is not clear about the coordination of gut commensal bacteria and SLE metabolism. Here, by integrating 16S sequencing and metabolomics data, we characterized the gut microbiome and fecal and serum metabolome alterations in patients with SLE. Microbial diversity sequencing revealed gut microflora dysbiosis in SLE patients with significantly increased beta diversity. The metabolomics profiling identified 43 and 55 significantly changed metabolites in serum and feces samples in SLE patients. Notably, lipids accounted for about 65% altered metabolites in serum, highlighted the disruption of lipid metabolism. Integrated correlation analysis provided a link between the gut microbiome and lipid metabolism in patients with SLE, particularly according to regulate the conversion of primary bile acids to secondary bile acids. Overall, our results illustrate the perturbation of the gut microbiome and metabolome in SLE patients which may facilitate the development of new SLE interventions.

## Introduction

Systemic lupus erythematosus (SLE) is a multifactorial autoimmune disease that can cause the damage of many organs and has a global prevalence. The manifestations of SLE are very diverse, and are related to immune system defects and the subsequent systemic inflammation (1). SLE is primarily treated with immunosuppressive drugs. However, there's still no effective approach that can cure SLE, and some people with SLE will ultimately die from other active diseases, such as kidney failure (2, 3).

The causes of SLE are still not fully understood, but are reported to be linked to many factors, including genetics, hormones and environmental factors (4). Those intrinsic and extrinsic factors act together and eventually will induce a systematic change of cellular metabolism. Recent studies had shown that the abnormal metabolic activities are critical in SLE pathogenesis. For example, glycolysis and mitochondrial oxidative metabolism are elevated in SLE patients and SLE mouse model, and specific inhibitors targeting these two metabolic pathways normalize the extreme activation of T cells in subjects with SLE and reduce the expression of SLE biomarkers (5). In addition to the metabolic signaling pathways, accumulation of T cell plasma membrane glycosphingolipids and cholesterol, which function as the structural molecules, exhibit pathogenic potential by increasing T cell signaling in SLE patients (6, 7). Moreover, the accelerated differentiation of B cells to plasma cells that leads to the overproduction of autoantibodies in SLE patients can be driven by an abnormal activation of mTORC1 signaling (8), indicating a close relationship between SLE and metabolism. Furthermore, metabolic changes in the body fluids, such as serum and urine, are also evident in SLE patients (9–11). Those reports suggest that the specific or global metabolic alterations observed in SLE patients may confer the SLE phenotypes, and more changes are still required to be uncovered.

Gut microbiome, which is considered as a new metabolic organ, is involved in regulating host metabolism. Their composition and abundance can be varied, depending on internal factors (e.g., host genetics) and external factors (e.g., diet, lifestyle, and drugs) (12). The change of gut microbiome composition has been reported to modulate the progression of many human diseases, such as non-alcoholic fatty liver disease, type 2 diabetes, and cancer (13). Accumulating evidence had proved that metabolites reside at the important interface between gut microbiome and the host health status (13–15). In SLE, gut microbiota alterations have been observed, and the association between microbiota and the pathogenesis of SLE have placed those small organisms as a critical focus in SLE research (16–19). However, the relationship between the gut microbiome and metabolism in SLE patients has not been determined. A comprehensive analysis of the gut microbiome and metabolome might therefore be helpful for us to uncover the complexity of SLE.

Here, we performed 16S gut microbiome sequencing and metabolomics studies in patients with SLE and healthy volunteers. We revealed a disruption of gut microbiome homeostasis and change of systemic metabolism in SLE patients. Furthermore, we also established a map showing the correlations between the gut microbiota and serum lipids. These results highlight the role of the gut microbiota in regulating host lipid metabolism and provide new insights into the pathological mechanisms of SLE.

## Materials and Methods

### Patient Information and Sample Collection

A total of 21 patients diagnosed as SLE according to the 1997 American College of Rheumatology classification criteria and 10 healthy volunteers were recruited for this study from January 2019 to June 2019 at Shenzhen People's Hospital (Guangzhou Province, China). The patients' characteristics, including age, gender, routine blood test results, autoantibodies test results (anti-nuclear antibodies, anti-ENA antibodies, anti-phospholipid antibody), immunoglobin, serum complement and liver biochemistry test results were recorded. The exclusion criteria were as follows: volunteers with other autoimmune diseases, such as rheumatic arthritis, systemic sclerosis, ankylosing spondylitis and Sjogren's syndrome; patients who received any anti-inflammatory treatment within 1 month before sampling; all participants including healthy and SLE patients who received antibiotics or probiotics treatment in 1 month; all participants with smoking or drinking habits, and all participants who had eaten fermented food, such as kimchi, within 1 week. All healthy participants were the same gender with SLE patients (female). They also had comparable age with SLE patients. None of the healthy volunteers had a history of any autoimmune diseases. All samples and clinical information were obtained under the condition of informed consent. This study was conducted with the approval of the Institutional Review Board of Shenzhen People's Hospital and in accordance with the Declaration of Helsinki.

The samples, including stool and serum from the same volunteer, were collected at the same day. Stool samples were self-collected after defecation at hospital and immediately placed on dry ice. Then the samples were transferred to the laboratory, divided into two parts and put into two frozen tubes. Blood samples were taken after an overnight starvation. After stratifying, the samples were centrifuged at 3,000 rpm for 10 min at room temperature. The supernatants were collected and transferred to two tubes. Any serum samples with even a little hemolysis were excluded in this study. All the stool and serum samples were put into liquid nitrogen for 15 min once collected, then transferred to −80°C for long term storage.

### Fecal DNA Extraction and 16S Sequencing

Genomic DNA from fecal samples were extracted by using the Mobio Powersoil DNA Isolation Kit (Qiagen, Hilden, Germany) according the manufacturer's instruction. The 16S rRNAs were amplified by using the primers (F-primer: 5′- ACTCCTACGGGAGGCAGCA-3′ and R-primer: 5′- GGACTACHVGGGTWTCTAAT-3′) targeting the V3 and V4 regions of 16S rRNA gene. After purification of the PCR product by using AMpure XP magnetic beads (Beckman Coulter, Indiana, USA), the samples were analyzed by Illumina HiSeq platform (Illumina, California, USA) through the paired-end sequencing strategy.

### Sequencing Data Analysis

After Illumina sequencing, paired-end reads were generated. According to the overlapping information of those reads, they were merged to form specific tags by FLASH software (Fast Length Adjustment of Short Reads, v1.2.7). Then we applied the Trimmomatic software (v0.33) to remove the low-quality tags and obtained ~75,000 clean tags from each sample. Clean tags were further filtered to exclude the chimeric sequences by UCHIME software (v4.2). Next, the remaining sequences (effective tags) with a similarity >97% were classified as an operational taxonomic unit (OTU). Taxonomic information was annotated by searching against the Silva database (www.Arb-silva.de), and OTUs were further assigned to different phylogenetic levels. The rarefaction curve was generated by the software QIIME (v1.8.0) based on the effective tags. Alpha diversity was calculated by Mothur (v1.30) to assess the bacteria richness and diversity of each sample. Beta diversity comparing the bacteria diversity between samples were calculated using QIIME software and the principal coordinates analysis (PCoA) was conducted with the R programming language (v3.5.1). The relative abundance and the difference in diversity were compared by Wilcoxon ran-sum test. Furthermore, linear discriminant analysis coupled with effect size (LEfSe) was applied to identify the microorganism that can discriminate SLE patients from healthy people.

### Sample Preparation for Metabolomics Study

One hundred μL serum from each sample were mixed with 300 μL methanol containing 1 μg/mL 2-chloro-L-phenylalanine (Hengbai Biotech, Shanghai, China) as the internal standard. After brief sonication in water bath at 4°C, all samples were placed in −20°C for 1 h. Then all samples were subjected to centrifugation at 12,000 rpm for 15 min at 4°C. For stool sample pretreatment, 50 mg of stool from each sample were put in a centrifuge tube, and then mixed with 1 mL extraction solution [methanol: acetonitrile: water = 2: 2: 1 (V/V/V)] containing 1 μg/ml 2-chloro-L-phenylalanine as the internal standard. After 30 s of vortex, all the samples were subjected to homogenization at 45 Hz for 5 min and then sonication for 5 min at 4°c. subsequently, samples were centrifuged at 12,000 rpm for 15 min at 4°C. For quality control (QC) sample preparation, an equal volume (10 μl) of each serum sample or stool extract were mixed together. The supernatants were then subjected to metabolomics profile analysis by ultra-high-performance liquid chromatography-mass spectrometry (UHPLC-MS).

### LC-MS Metabolomics Data Acquisition

All the serum and feces samples were analyzed by 1290 UHPLC (Agilent Technologies, California, USA) coupled with Thermo Q Exactive Focus (Thermo Fisher Scientific, Massachusetts, USA) platform by Biotree Ltd. (Shanghai, China). Briefly, samples were analyzed in both positive and negative ion mode. Mobile phase A used in positive ion mode was 0.1% formic acid in water, and in negative mode it was 5 mmol/L ammonium acetate in water. Mobile phase B was acetonitrile. The elution gradient was set as follows: 1% B at 1 min, 99% B at 8 min, 99% B at 10 min, and 1% B at 10.1 min, 1% B at 12 min. The flow rate was set to 0.5 mL/min. The Q Exactive mass spectrometer was run at the spray voltage of 4.0 kV in positive mode and −3.6 kV in negative mode, respectively. Other ESI source conditions were as follows: sheath gas flow rate of 45 Arb, Aux gas flow rate of 15 Arb, and capillary temperature of 400°C. All the MS1 and MS2 data were obtained in the control of Xcalibur (Thermo Scientific). UPLC HSS T3 column (Waters, Massachusetts, USA) was used for all analysis. The organic reagents, including methanol, acetonitrile and formic acid (HPLC grade) were purchased from CNW technologies (Dusseldorf, Germany).

### LC-MS Metabolomics Data Analysis

The acquired MS raw data were transformed to mzXML format by using ProteoWizard software, and processed by XCMS. The data preprocessing steps include peak identification, peak alignment, peak extraction, retention time correction and peak integration. To make the metabolomics data reproducible and reliable, the relative standard deviation (RSD) of the peaks >30% in the QC samples were filtered out. The remaining peaks were identified by comparison to retention time and mass to charge ratio (m/z) indexes in the library consisting of information from the online database of HMDB (www. hmdb.ca), KEGG (www.genome.jp/kegg) and the in-house library. Peak intensity was quantified by using the area under the curve. After that, we obtained a three-dimensional data matrix consisting of the retention time values, m/z values and peak intensities. The data matrix was further processed by removing the peaks with missing values in more than 50% samples and filling up the left missing values with half of minimum value. Then, a new data matrix was generated by normalizing the data to the peak intensity of the internal standard. To understand the difference of metabolomics profile between SLE patients and healthy people, the multivariate statistical analyses, including principal component analysis (PCA) and orthogonal projections to latent structure-discriminant analysis (OPLS-DA), were carried out. At the same time, student's *t*-test was used to identify the altered metabolites in SLE patients. The metabolites with a VIP value>1 (variable importance in the projection) in OPLS-DA analysis and *P* < 0.05 in univariate analysis were considered as significantly changed metabolites.

### Statistical Analysis

Statistical analysis was performed by using Microsoft Excel (Microsoft Inc., Redmond, Washington) and R software version 3.5.1 (R Foundation for Statistical Computing, Vienna, Austria). The differential abundance of bacterial taxa at different levels (phylum, class, order, family and genus) between SLE patients and healthy controls were calculated by Wilcoxon rank-sum test. The differences of alpha diversity, including OTU number, Shannon index, Simpson index and Chao1 index, were determined by student's *t*-test. Permutational multivariate analysis of variance (PERMANOVA) and analysis of similarity (ANOSIM) analysis were conducted to assess the difference of beta diversity between SLE and control group. The significantly altered metabolite class in fecal and serum samples was tested by Fisher's exact test. Spearman's correlation was used to describe the relationships between the gut microbiota and altered lipid species and between the gut microbiota and the well-known gut microbiota metabolites. The specific correlation map was drawn by using Cytoscape (20). To predict SLEDAI score with bile acids, multiple linear regression was used. Differences were considered significant when *p* < 0.05, and the data were presented as mean ± standard error (mean ± se).

## Results

### Characterization of Participants

In total, 10 healthy controls and 21 SLE volunteers were included in this study. Since SLE is more prevalent in women (women: men = 9: 1), we only recruited women in this analysis. The two groups had comparable age of about 37 years (SLE, 37.48 ± 2.44 years, healthy controls, 37.50 ± 3.02 years, data are mean ± se). The basic screen of the participants showed that blood lipids, including cholesterol and triglycerides (TG), had no significant difference between the two groups (Figure S1). Besides, the level of Alanine Aminotransferase (ALT) and Aspartate Aminotransferase (AST) that reflecting liver function were in the normal range (7–55 U/L for ALT and 8–48 U/L for AST according to the guidelines from the Mayo Clinic), though AST in SLE patients (17 ± 1.21 U/L) was significantly smaller than healthy controls (22.6 ±1.09 U/L) with a *p-*value of 0.045 by student's *t*-test (Figure S1). Furthermore, the level of serum creatinine (SCr) that reflecting kidney function were also comparable between the two groups (Figure S1). Detailed descriptions of SLE classification tests, such as anti-ANA, anti-ENA and complement tests, and SLEDAI (systemic lupus erythematosus disease activity) score were shown in Table S1.

### Altered Gut Microbiota Diversity in SLE Patients

**Figure 1:**
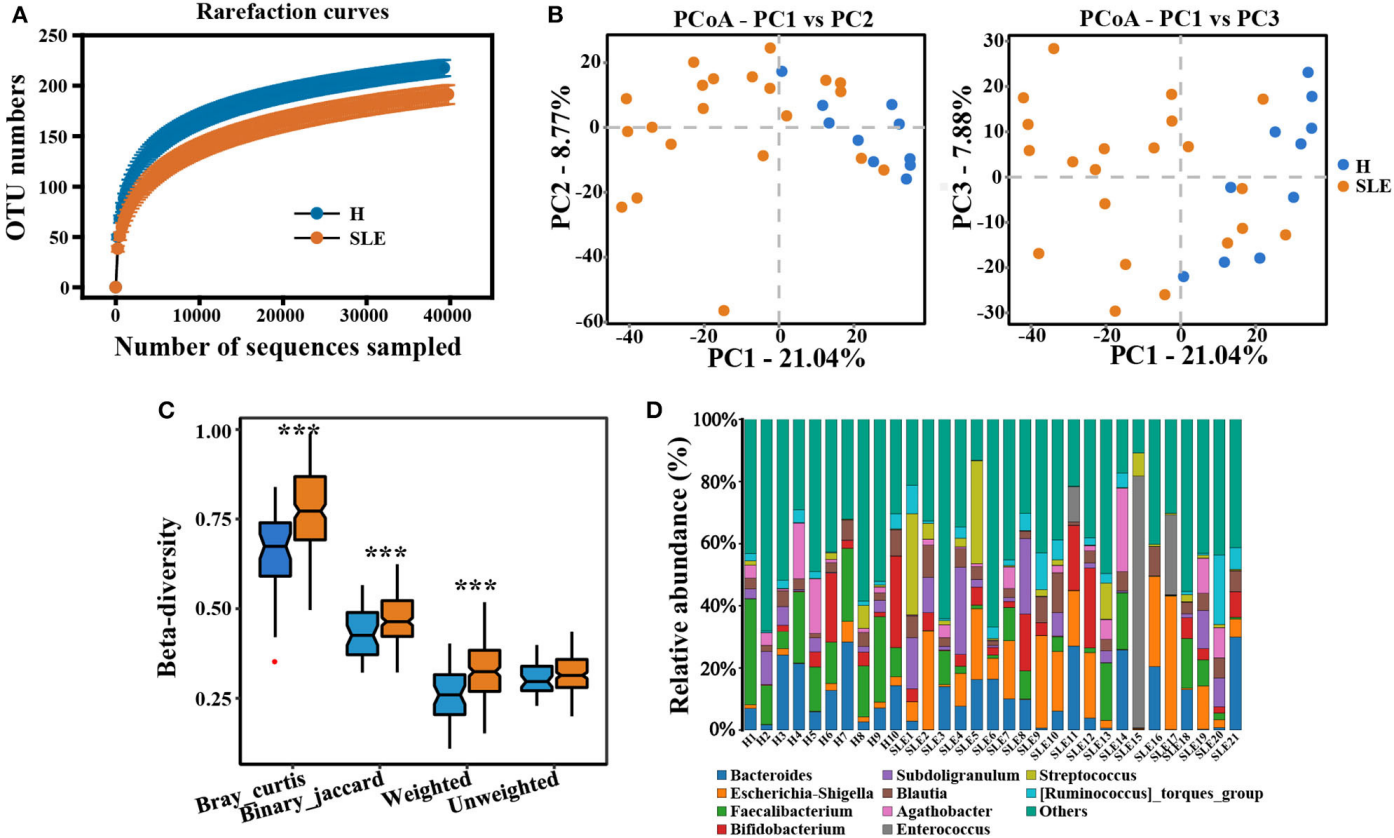
Altered gut microbiota diversity in SLE patients. **(A)** Rarefaction curve based on OUT count in healthy controls and SLE patients. H, healthy people (orange); SLE, SLE patients (blue). **(B)** The PCoA ordination of Bray-Curtis distances between SLE patients and healthy controls from 16S rDNA sequencing data. The first three axes of PCoA showed a clear separation. Left, PCoA (PC1-PC2); Right, PCoA (PC1-PC3). **(C)** Beta diversity index calculated by bray_curtis distance, binary_jaccard distance, weighted distance and unweighted distance. Student's *t*-test or rank-sum test. ***p < 0.001. **(D)** The relative abundance of dominant taxa at genus level in each sample.

In the present study, a total of 1,817,156 effective 16S sequencing tags were obtained from the stool samples of 10 healthy controls and 21 SLE patients, with an average of 58,617.9 per sample (ranging from 45,625 to 67,825). After taxonomic identification and very low abundant taxonomies filtering (lower than 0.005%), 400 operational taxonomic units (OTUs) were left for further analysis (Table S2). According to rarefaction curve (Figure 1A) and species accumulation curve (Figure S2A), the current sequencing and samples were sufficient for taxa identification. In addition, the rank abundance distribution curve showed decreased richness and evenness of the bacteria in SLE patients (Figure S2B). We then compared the microbial differences between healthy controls and SLE individuals. Consistent with the reports by Zhixing He et al. in Chinese populations (12), we also found the lowered OTU counts in SLE patients than controls (Figure 1A, Figure S2C). However, alpha diversity indexes, including Shannon index, Simpson index and Chao1 index, in SLE patients were not significantly altered comparing with control group (Figure S2). Difference in beta diversity between the two groups were observed by PCoA analysis of Bray-Curtis distance (Figure 1B), which was significant according to PERMANOVA (*p* = 0.001) and ANOSIM analysis (*p* = 0.023). Besides, the bray_curtis, binary_jaccard and weighted distance were significantly increased in SLE patients (Figure 1C), confirmed the alteration of microbiome community structure in SLE patients.

**Figure 2:**
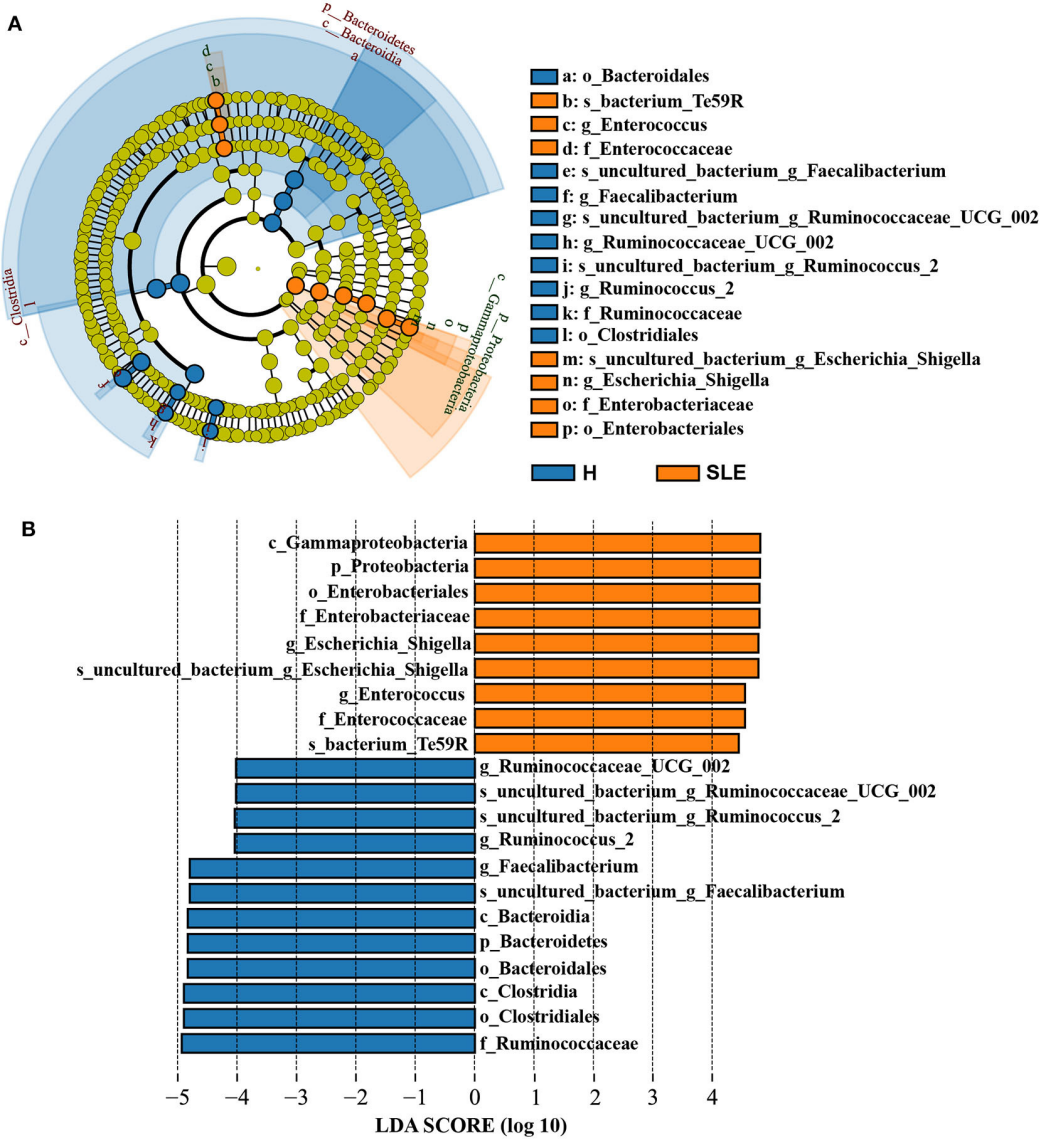
Linear discriminant analysis (LDA) Effect size analysis. **(A)** Cladogram of LEfSe linear discriminant analysis of microbiome from 16S rDNA sequencing of Control and SLE patients. Orange and blue circles represent the differences of the most abundant microbiome class. The diameter of each circle is proportional to the relative abundance of the taxon. **(B)** Histogram of the LDA scores for different abundant genera in healthy control and SLE patients. Orange, enriched in SLE patients; Blue, enriched in healthy controls.

In addition, our data showed a considerable difference in the relative abundance of each bacterium at genus level across samples in SLE patients and healthy people (Figure 1D, Figure S3). To find out the alterations associated with SLE, we conducted LEfSe analysis. The main differences were the reduction in abundance of *Bacteroidetes* (class *Bacteroidia* and order *Bacteridales*) and the increase of *Proteobacteria* (class *gammaproteobacteria*, order *Enterobacteriales* and family *Enterobacteriaceae*) in SLE patients (Figure 2A). Some differences were also observed at a lower taxonomical level. SLE patients showed an increment of *Enterococcaceae* (genus *Enterococcus*) and *Escherichia_Shigella*. By contrast, SLE patients exhibited a loss of *Clostridia* in class level (order *Clostridiales*), *Ruminococcaceae* in family level (*Ruminococcus_2* and *Ruminococcus_UCG_002* in genus level) and *Faecalibacterium* in genus level (Figure 2B, Figure S3). Taken together, those data indicate the alteration of commensal gut microbiome composition in SLE patients, suggesting a dysregulation of the microbial community.

### Serum and Fecal Metabolite Profiling in SLE Patients

**Figure 3:**
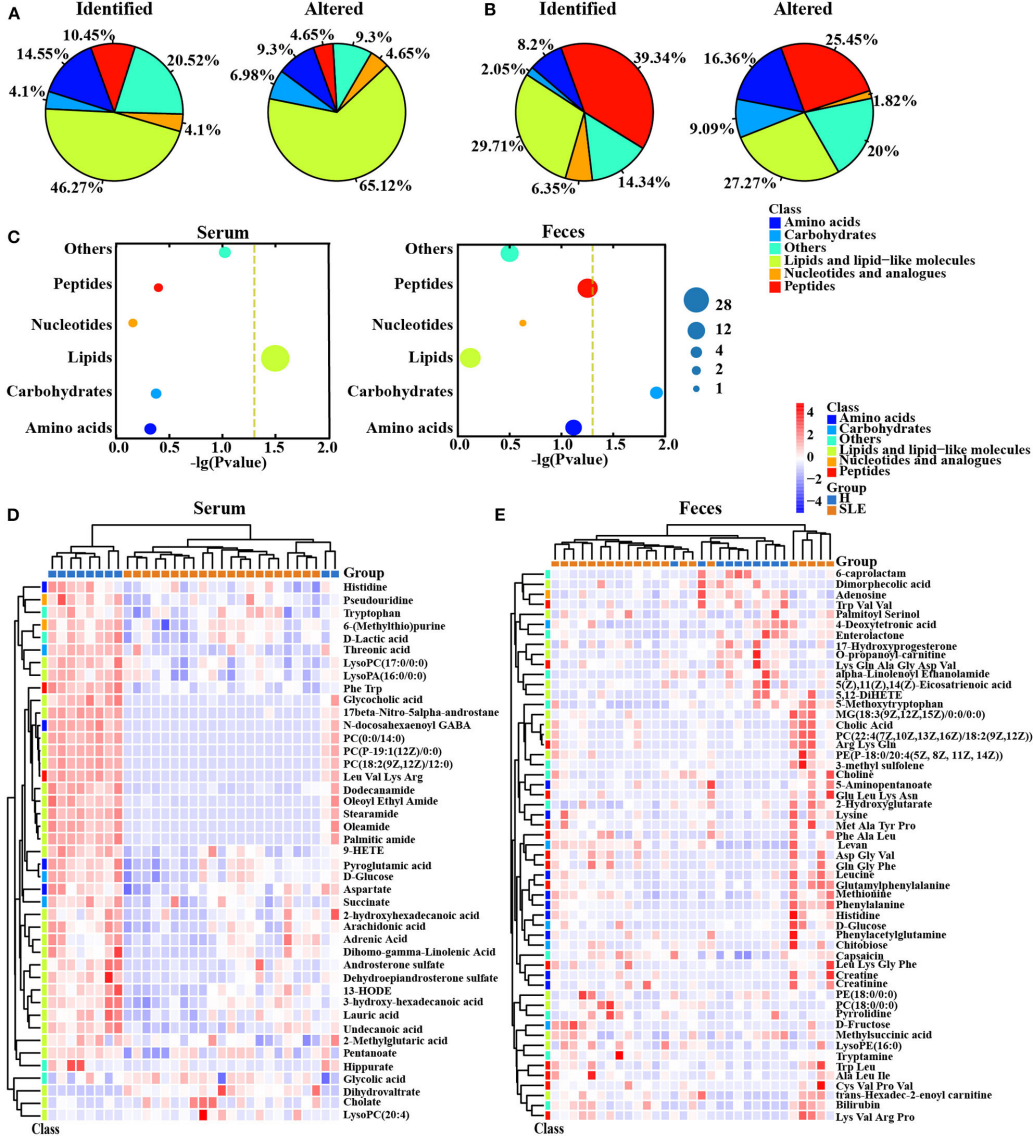
Serum and feces metabolic profiling in SLE patients. **(A)** Pie graph of the metabolite class composition of identified and significantly altered metabolites in SLE patients according to the number of metabolites in serum. Left, identified serum metabolite class composition; Right, significantly altered serum metabolite class composition. **(B)** Pie graph of the metabolite class composition of identified and significantly altered metabolites in SLE patients according to the number of metabolites in feces. Left, identified fecal metabolite class composition; Right, significantly altered fecal metabolite class composition. **(C)** Enrichment analysis of significantly changed metabolite class in serum and feces samples. The size of each dot represents the number of metabolites significantly changed in the corresponding metabolite class. The dashed yellow line represents the significant level (*p* = 0.05). **(D)** Significantly changed metabolite heatmap in serum samples. The abundance of each metabolite is normalized by Z score normalization. Metabolite class is shown in the left of the heatmap. **(E)** Heatmap for significantly changed metabolites in feces samples. The abundance of each metabolite is normalized by Z score normalization. Metabolite class is shown in the left of the heatmap.

Many papers have reported the correlation between the composition of gut microbiota and tissue metabolome (14, 15), thus we performed metabolomics profiling study in the serum and feces samples to uncover the metabolome alterations in SLE patients. Hundreds of metabolites were identified by using liquid chromatography-mass spectrometry (LC-MS), with 268 in serum and 488 in feces (Tables S3, S4). All metabolites were classified to several classes, including Amino Acids, Peptides, Lipids, Carbohydrates, Nucleotides and Others. In both serum and feces, lipids accounted for a major proportion of metabolites identified. In contrast to the serum metabolite profile, many peptides were also identified in feces samples (Figure 3B). Besides, the impact of SLE on the level of metabolites in serum and feces is varied (Figures 3A,B). Although more metabolites identified in feces, the percentage of significantly altered metabolites in the serum samples was greater than in feces samples (~16.0% significantly changed in serum vs. 11.3% in feces) (Figure S4A, Tables S5, S6). In addition, lipids accounted for a large part of significantly changed metabolites in both serum and feces. Especially in serum, the identified lipids accounted for about 46.3% of total identified metabolites, but the altered lipids in SLE patients were more than 65% of all changed metabolites (Figure 3A) and were significantly enriched (*p* = 0.0318 according to Fisher's exact test, Figure 3C and Table S7), suggesting a disruption of lipid homeostasis in SLE patients.

The differentially changed metabolites in serum and feces are presented in Figures 3C,D, respectively. Notably, a very interesting characteristic in serum is the decrease of almost all of the altered metabolites, including many membrane structural lipids and free fatty acids. However, in feces, except some lipids, metabolites of amino acids and peptides are increased in SLE patients, suggesting a possible effect of bacterial proteolytic fermentation. Furthermore, KEGG metabolic pathway mapping showed that the significantly altered metabolites were mapped to about 30 pathways, including amino acid metabolism and lipid metabolism pathways (Figures S4B,C). Clearly, some differences existed between the altered metabolic pathways in serum and feces. Those distinct metabolic profiles between serum and feces suggest different impact on SLE pathogenesis.

### Metabolic Network Disruption in SLE Patients

**Figure 4:**
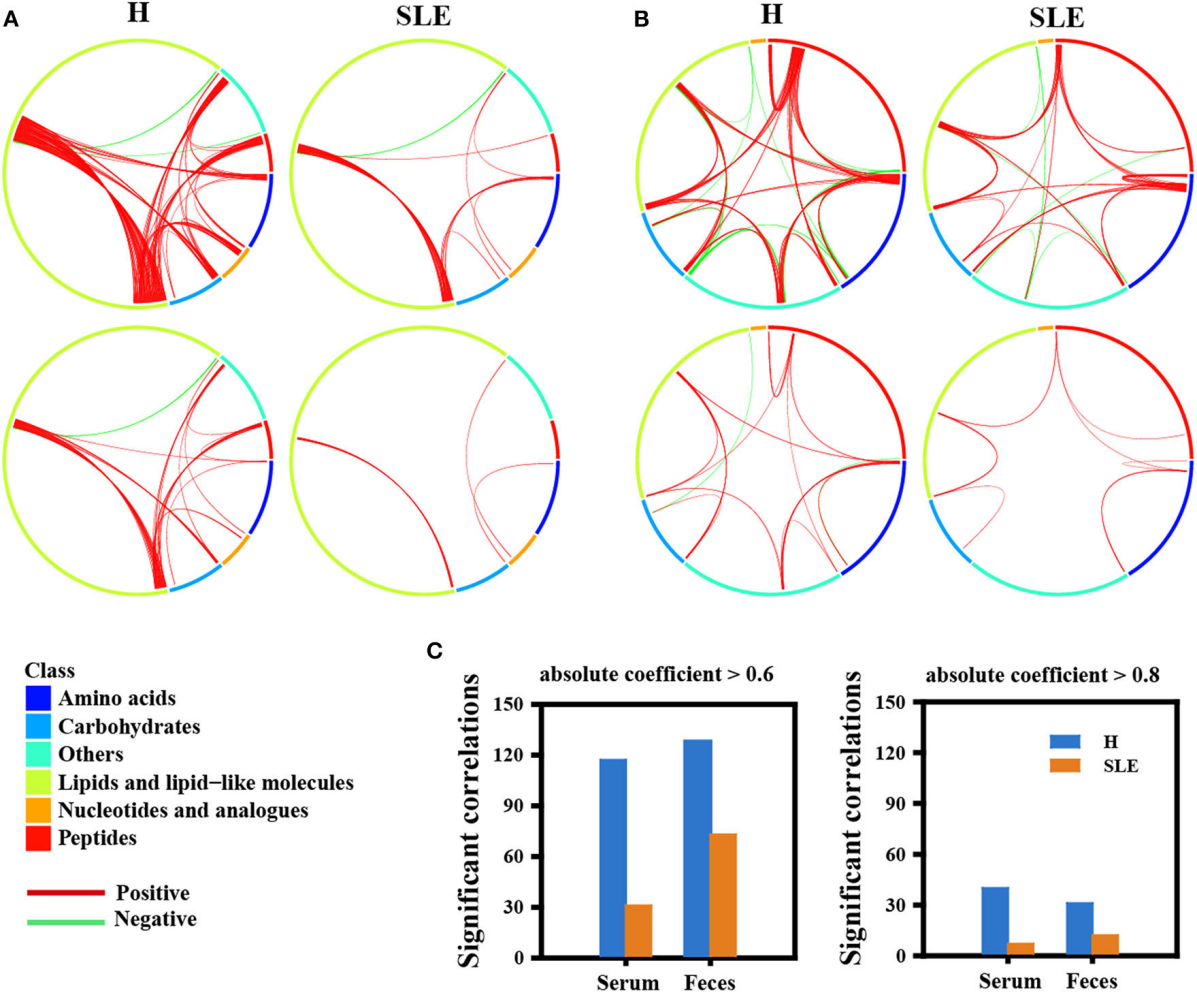
Metabolic network disruption in SLE patients. **(A)** ChordDiagram showed the significant correlations between the significantly altered metabolites of different class or within the same class in serum. Metabolite class was shown as the color bar around the circumference. Each line indicates a significant correlation (spearman's correlation, *p* < 0.05). Red, positive correlation; Cyan, negative correlation. Upper, absolute coefficient >0.6; Lower, absolute coefficient >0.8. **(B)**. ChordDiagram showed the significant correlations between the significantly altered metabolites of different class or within the same class in feces. Metabolite class was shown as the color bar around the circumference. Each line indicates a significant correlation (spearman's correlation, *p* < 0.05). Red, positive correlation; Cyan, negative correlation. Upper, absolute coefficient >0.6; Lower, absolute coefficient >0.8. **(C)** Statistic results of No. of significant correlations in serum and feces in healthy people and SLE patients.

Metabolic correlations imply the coordination of different cells within the tissue, and the maintenance of metabolic relationships is essential for tissue homeostasis (21). We hypothesized that the metabolic interaction network in SLE patients is disrupted, since multi-organ abnormalities are a common feature of this disease. We analyzed the linear correlations of altered metabolites in healthy volunteers and SLE patients, respectively (Table S8). Firstly, all significant correlations (*p* < 0.05) in healthy controls had an absolute correlation coefficient >0.6 (|*r*| > 0.6), namely 117 pairs in serum samples and 128 in fecal samples. However, only 31 correlations in serum and 73 in feces of SLE patients presented a correlation of |*r*| > 0.6. Among them, 40 pairs scored with high correlations (|*r*| > 0.8) in the serum of the control group compared with 7 in SLE patients (Figures 4A,C). A very similar phenomenon was also observed in the feces of SLE patients, with about 50% loss of the highly collinear metabolite pairs compared with healthy controls (Figures 4B,C). The substantial loss of metabolite correlations in SLE patients implies a disruption of whole-body homeostasis, which may lead to much more severe damage in the presence of additional etiological factors.

Further examination of the correlations, we found that the global correlations in serum reduced severely in SLE patients, especially the correlation of lipids with other metabolites. Importantly, relationships of lipids with carbohydrates and nucleotides were lost in patients with SLE. Some correlations between metabolite classes were maintained (Figure 3A). In addition, similar results were also obtained in feces (Figure 3B). The global loss of correlations suggests a loss of systematic metabolic integrity of the whole body in SLE patients.

### Gut Microbiota Metabolites Alteration in The Serum of SLE Patients

**Figure 5:**
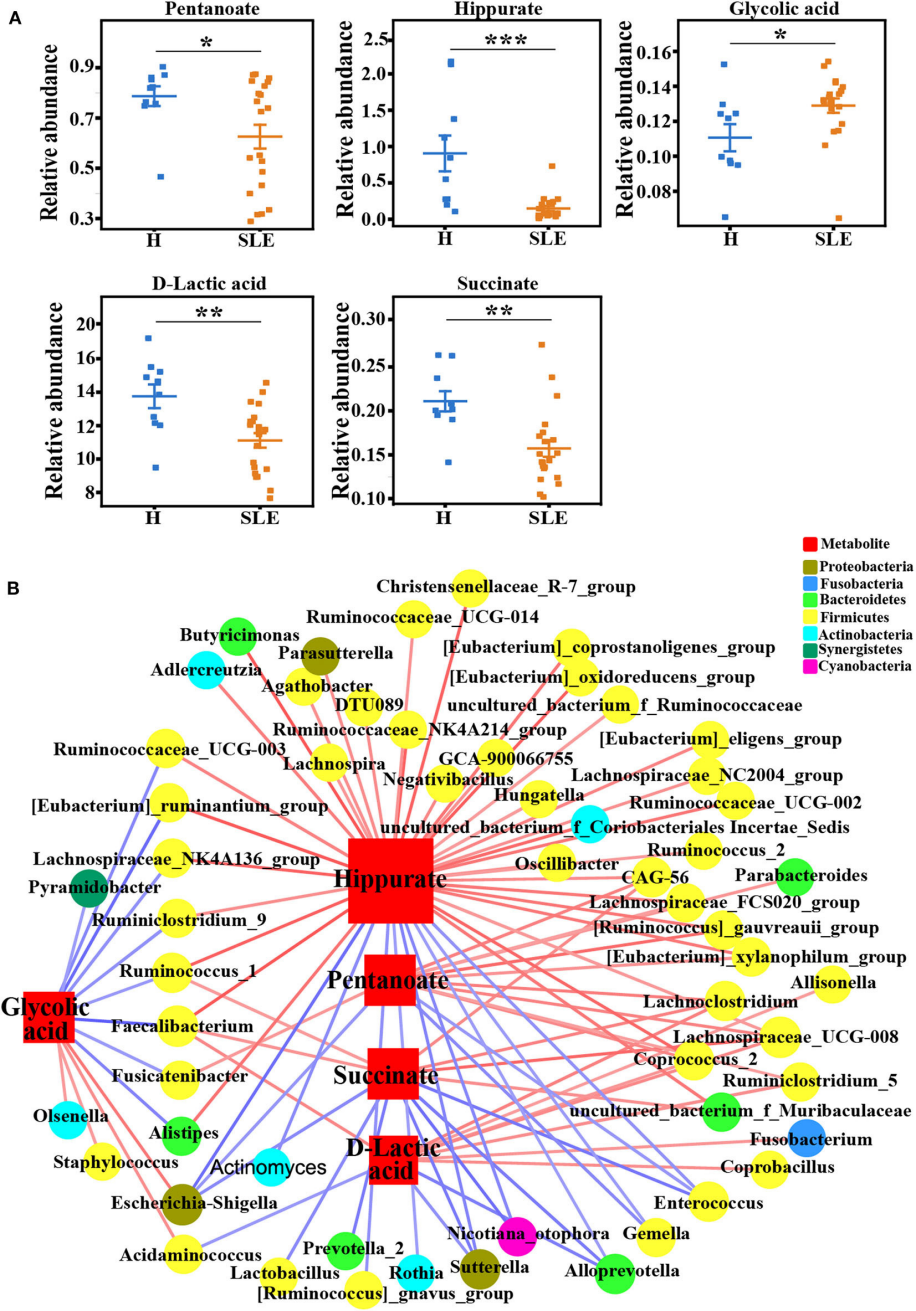
Gut microbiome metabolites alteration in the serum of SLE patients. **(A)** The relative abundance of Pentanoate, Succinate, D-lactic acid, Hippurate and Glycolic acid in the serum of healthy people and SLE patients. Data represents mean ± se. Student's *t*-test or rank-sum test. *p < 0.05; **p < 0.01; ***p < 0.001. **(B)** The correlation network of gut microbiome and gut microbiome metabolites in **(A)**. Square indicates metabolites, and the size of square represents the difference between SLE and control group. Circle represents different bacterial in genus level. Color of the circles represents different bacterial phylum. Lines indicates significant correlation (spearman's correlation, *p* < 0.05 and absolute coefficient >0.3) between a metabolite and a bacterium in genus level. Red line, positive correlation; Blue line, negative correlation.

In our metabolomics analysis, many well-known gut microbiota metabolites were identified, including SCFAs, indoles, phenols, and bile acids. Several of these metabolites were significantly changed in serum samples of SLE patients (Figure 5A). Pentanoate, a short chain fatty acid (SCFA) exhibiting the ability of inhibiting autoimmunity (21), was significantly decreased in SLE patients. In addition, the levels of D-lactic acid, succinate, as well as hippurate that were reported to be the product of microbial carbohydrate fermentation (13), were also decreased (Figure 5A). Furthermore, glycolic acid, which showed the ability to induce kidney toxicity in previous report (22), was significantly increased. Those results suggest that the disruption of microbial metabolism may contribute to the pathogenesis of SLE.

As the levels of the aforementioned metabolites were significantly altered in SLE patients, we searched for genera in the gut microbiota that might correlate with them (Table S9). Most of the correlations were established with the microorganisms from *Firmicutes* (phylum, yellow circles) (Figure 5B). Besides, we noticed that some bacteria that positively correlated with hippurate, pentanoate, succinate and lactic acid, including *Fusicatenibacter, Faecalibacterium, Ruminococcaceae_UCG-003, Ruminococcus_1, Alistipes, [Eubacterium]_ruminantium_group, Lachnospiraceae_NK4A136_group* and *Ruminoclostridium_9*, were negatively correlated with glycolic acid. Interestingly, except *Alistipes*, other species are all from *Firmicutes* (phylum)_*Clostridia* (class)_*Clostridiales* (order) taxonomy. *Escherichia-Shigella* that was enriched in SLE patients (Figure 2B) was positively correlated with glycolic acid. Interestingly, *Clostridia* had been reported to attenuate human immune disorders (23), and *Escherichia-Shigella*, a well-known pathogen that belongs to the *Proteobacteria* (phylum) and *Gammaproteobacteria* (class), has been reported to modulate innate and adaptive immune responses (24). These changes of gut commensal bacteria and their metabolites imply a coordinated effect and a contribution to SLE pathogenesis.

### Global Correlation of The Microbiome and Altered Lipid Metabolism

**Figure 6:**
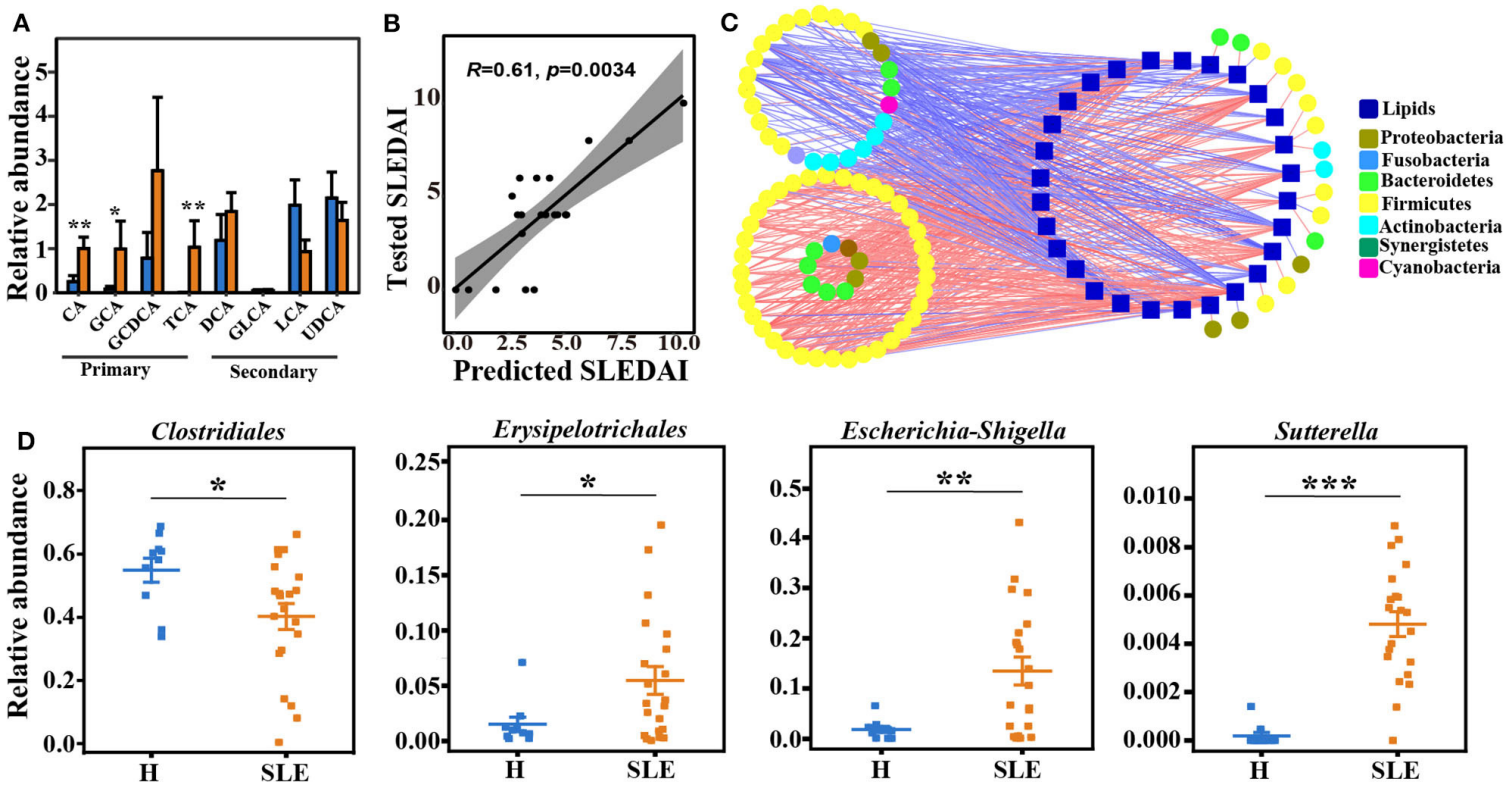
Global correlation of gut microbiota and altered serum lipids. **(A)** Relative abundance of fecal bile acids of SLE patients and healthy controls. Data represents mean ± se. Student's *t*-test or rank-sum test. *p < 0.05; **p < 0.01. **(B)** Multiple linear regression model to predict SLEDAI score by fecal bile acids. **(C)** The correlation network of gut microbiome and altered serum lipid species. Square indicates lipid species. Circle represents different bacteria in genus level, and the color of circle indicates different phylum. Lines indicates significant correlation (spearman's correlation, *p* < 0.05 and absolute coefficient >0.3) between a lipid metabolite and a bacterium in genus level. Red line, positive correlation; Blue line, negative correlation. **(D)** Relative abundance of the selected bacteria that correlated with bile acids and lipid metabolism. Data represents mean ± se. Student's *t*-test or rank-sum test. *p < 0.05; **p < 0.01; ***p < 0.001.

The metabolism mediated by the gut microbiota is very important for the shaping of human metabolic profile and is tightly related to human health. We analyzed the total levels of well-known microbiota metabolites in the feces and serum to determine potential changes of different types of gut microbiota metabolites. Interestingly, the levels of primary bile acids, including cholic acid (CA), glycocolic acid (GCA), taurocholic acid (TCA) and glycochenodeoxycholic acid (GCDCA), were increased in feces from SLE patients (Figure 6A). Gut commensal bacteria are responsible for the conversion of primary bile acids to secondary bile acids (25). In our data, a trend of decrease of secondary bile acids (DCA, deoxycholic acid; LCA, lithocholic acid; GLCA, glycolithocholic acid; UDCA, ursodeoxycholic acid) was observed in feces samples of SLE patients, though the difference were not significant (Figure 6A). Further analysis through a multiple linear regression model showed a good prediction of SLEDAI score (*R* = 0.61, *p* = 0.0034) by fecal bile acids (Figure 6B), demonstrating a correlation of bile acids with SLE activity.

It is worth to note that the conversion of primary bile acids to secondary bile acids by bacteria is critical for the progression of some diseases as reported (26, 27), and bile acids are well-known for their lipid digestive function (28). We then analyzed the correlation of gut microbiome at genus level and altered lipids (Figure 6C and Table S10). The majority of the bacteria that correlated with those altered lipids belong to *Firmicutes* (phylum level, yellow circle). In this phylum, *Lactobacillales* and *Erysipelotrichales* are the taxa accounting for the negative correlations, while *Clostridiales* is mainly responsible for positive correlations. Notably, the relative abundances of *Clostridiales* and *Erysipelotrichales* were significantly changed in SLE patients (*p* < 0.05, Wilcoxon rank-sum test; Figure 6D). Other microorganisms, including *Bacteroidetes, Actinobacteria, Fusobacteria, Proteobacteria* and *Tenericutes*, are also positively related to those lipids. Additionally, *Proteobacteria*, which contains a wide variety of pathogens including *Escherichia-Shigella* and *Sutterella* (Figures 6C,D), are negatively correlated with those altered lipids. This finding is consistent with the role of *Escherichia-Shigella* in regulating lipid metabolism in colon cancer (29). Furthermore, *Bacteroides, Clostridium, Lactobacillus, Eubacterium, Eggerthella, and Ruminococcus* are directly involved in bile acid metabolism (30).

To further understand the relationship of gut microbiome, serum lipid metabolism and SLE activity, we performed spearman's correlation analysis. Most of the bacteria that significantly correlated with SLEDAI belong to *Firmicutes*, and are negatively correlated. Lipids that significantly correlated with SLEDAI are bile acids (deoxycholic acid, glycocholic acid, isohyodeoxycholic acid) and arachidonic acid (Figure S5). Our data suggest that the intestinal bacterial metabolism of bile acids may play an important role in regulating lipid homeostasis in SLE patients and are related to SLE activity.

## Discussion

Previous studies using integrative biological tools had revealed the alterations associated with SLE at the DNA, RNA and protein levels. However, it is not sufficient to understand the global changes occurring along with the systemic impairments of SLE patients. Here, we recruited 21 SLE patients, and investigated the gut microbiome alterations in feces and the metabolome changes in serum and feces. According to our results, metabolomics homeostasis is disrupted in SLE patients, especially lipid metabolism which is tightly correlated with the gut microbiome. In addition, we also found the alterations of several well-known gut microbiota produced metabolites that may affect the pathogenesis of SLE.

More and more evidence suggest that gut microbiome contributes to the pathogenesis of SLE (12, 16, 19). In the present study, the gut microbiome of SLE patients displayed lower bacterial alpha diversity and altered beta diversity, which is consistent with previous reports (12, 31), confirming the occurrence of dysbiosis. Among those identified microorganisms, some were found to be significantly altered in SLE patients. *Gammaproteobacteria* (class level) and the corresponding phylum *Proteobacteria* were significantly enriched in SLE group, which is similar with previous report (31). Interestingly, *Proteobacteria*, in particular the *Enterobacteriaceae* family that was also significantly increased in SLE patients, has been shown previously to be associated with intestinal inflammation (32), reflecting the common feature of inflammatory response in SLE patients. *Bacteroidetes* and *Firmicutes* are the two most abundant bacterial phyla in human gut. A previous study reported a decrease of *Firmicutes*/*Bacteroidetes* (F/B) ratio in SLE patients (33). While another research group showed that there's no alteration in the F/B ratio of SLE patients (31). In contrast to these two reports, our results revealed an ~1-fold increase in the F/B ratio in SLE patients, along with a significantly decrease in the abundance of *Bacteroidetes* (Figure S3A). In one aspect, the discrepancy is possibly due to the differences of the study populations, as the volunteers we recruited were all Chinese women with younger ages. In another aspect, the reduction of the abundance of *Bacteroidetes* has been implicated as critical factor contributing to the progression of obesity and other metabolic diseases (34). At the same time, the production of short chain fatty acids (SCFAs) by *Bacteroidetes* through the fermentation of fibers was reported to be beneficial to improve the disease status (13, 34). Our results demonstrated that the dysbiosis, including the decrease of potentially beneficial *Bacteroidetes* and the increase of the potentially pathological *Proteobacteria*, may contribute to the pathogenesis of SLE.

The metabolic alterations might be helpful to discover the underlying pathological mechanisms of SLE (35). Our metabolomics data showed different metabolism patterns between fecal and serum samples in SLE patients (Figure 3). In feces, half of the identified carbohydrates were significantly changed, while the impact of SLE on serum was mainly on lipid metabolism (Figure 3). A recent study described the fecal metabolic profiles in SLE patients and identified several significantly altered metabolites, including Methionine, Adenosine and LysoPE (16: 0), which were also significantly changed in our study (36). Besides, altered lipid profiles were also observed in previous studies (9–11). The homeostasis of a particular organ can be achieved by coordination of the metabolic network, and it can be potentially disrupted in disease condition (37). We observed an obvious loss of metabolite correlations in the serum and feces of SLE patients (Figure 4), suggesting a more fragile system that may lead to susceptibility to pathogens. Taken together with previous reports, our data further suggest that the alteration of fecal and serum metabolome could be helpful for understanding the pathogenesis of SLE.

Metabolites of gut microbiota, which are usually secreted in the intestine and translocated across the intestinal barrier into circulating system, are very important modulators for host metabolism (13, 28). In the serum samples, our results revealed several significantly changed gut microbiota metabolites (Figure 5). Among them, pentanoate, a short chain fatty acid, along with succinate and lactate, that have been shown by other scientists to be beneficial for the prevention and treatment of obesity (13), were significantly decreased. In contrast, glycolic acid, which was shown to be toxic to kidney (22), was increased in patients with SLE, demonstrating a potential pathological role of gut commensal bacterial metabolism in SLE and possible impairment of kidney function. In addition to those gut microbiota metabolites, lipids scored as the most significantly changed metabolite class in serum of SLE patients (Figure 3). Bile acids, a class of amphipathic molecules, are well-known for their lipid digestive function (28). However, the link among gut microbiota, bile acids and host lipid metabolism are not investigated in SLE. Our data revealed a defect of gut microbiome mediated transformation of bile acids and the potential influences on host lipid metabolism in SLE.

In summary, this study combining the fecal and serum metabolomics and fecal 16S sequencing data reveals the global metabolic homeostasis status and the relationship between gut microbiota and host lipid metabolism in SLE patients. Additional studies are needed to further address the exact bacterial species that paly the key role in SLE metabolism. Our data provides the underlying mechanism by which the gut microbiota regulates host metabolism in SLE patients, which will be helpful for providing new directions for further research and developing new rational drugs for therapy.

## Data Availability Statement

The 16S sequencing data has been uploaded to the European Nucleotide Archive under the project accession: PRJEB39044. Other raw data supporting the conclusions of this article will be made available by the authors, without undue reservation, to any qualified researcher.

## Ethics Statement

The studies involving human participants were reviewed and approved by The Institutional Review Board of Shenzhen People's Hospital. Written informed consent to participate in this study was provided by the participants' legal guardian/next of kin.

## Author Contributions

JH: conceptualization, methodology, formal analysis, data curation, investigation, visualization, and writing (original draft). TC: formal analysis, data curation, software, and visualization. XH: resources and data curation. FZ and CZ: resources. LY: supervision. WD: writing (review and editing). DT and DL: supervision and funding acquisition. YD: conceptualization, supervision, writing (review and editing), funding acquisition. All authors contributed to the article and approved the submitted version.

## Conflict of Interest

TC was employed by company Shanghai Biotree Biomedical Technology Co., LTD. The remaining authors declare that the research was conducted in the absence of any commercial or financial relationships that could be construed as a potential conflict of interest.
