# Supplementary figures and images for: Microbiome and Metabolome Analyses Reveal the Disruption of Lipid Metabolism in Systemic Lupus Erythematosus
Source: Front Immunol. 2020 Jul 31;11:1703. doi: 10.3389/fimmu.2020.01703 (PMC7411142; doi:10.3389/fimmu.2020.01703)

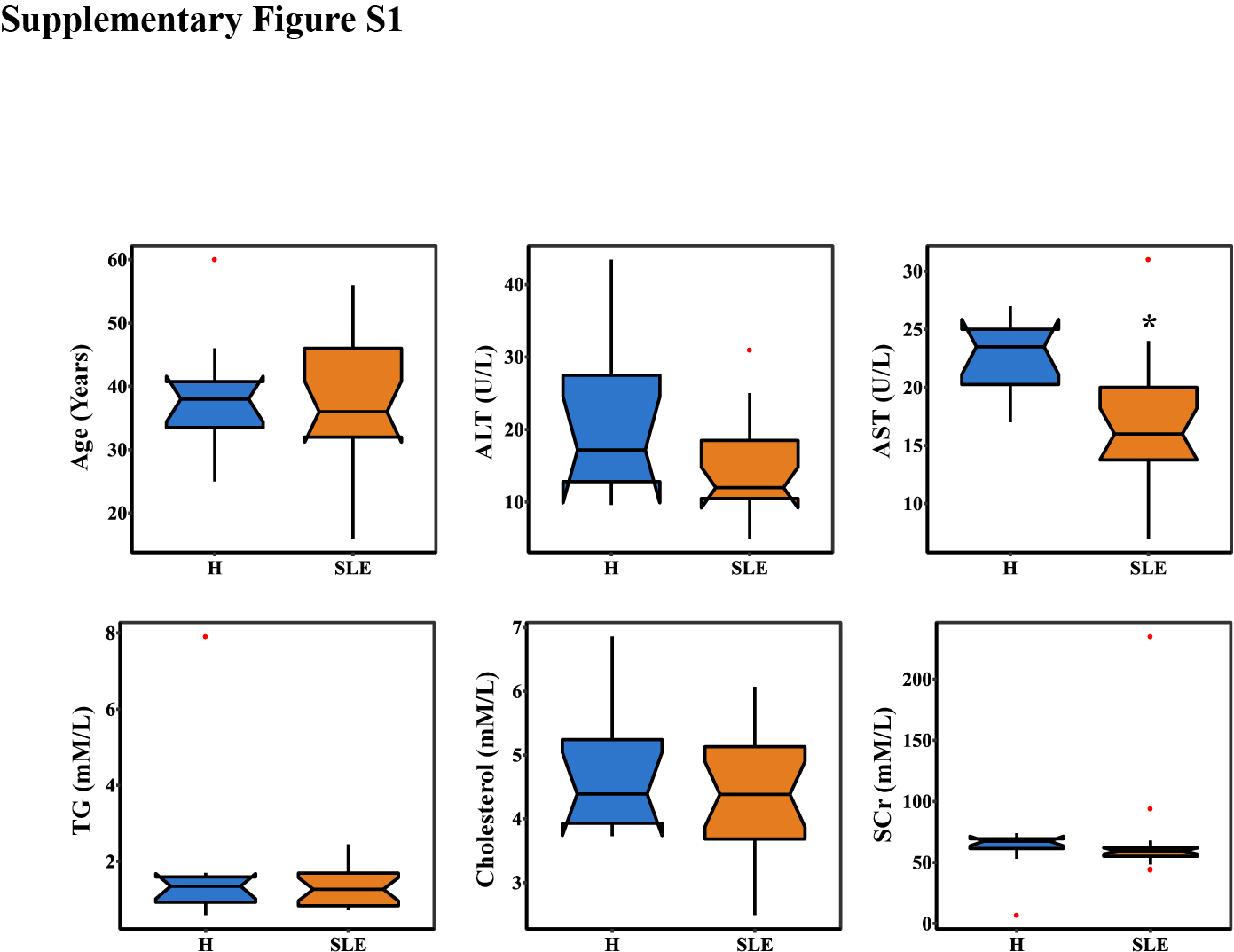

Supplement: Supplementary file 1 [file Image_1.JPEG]

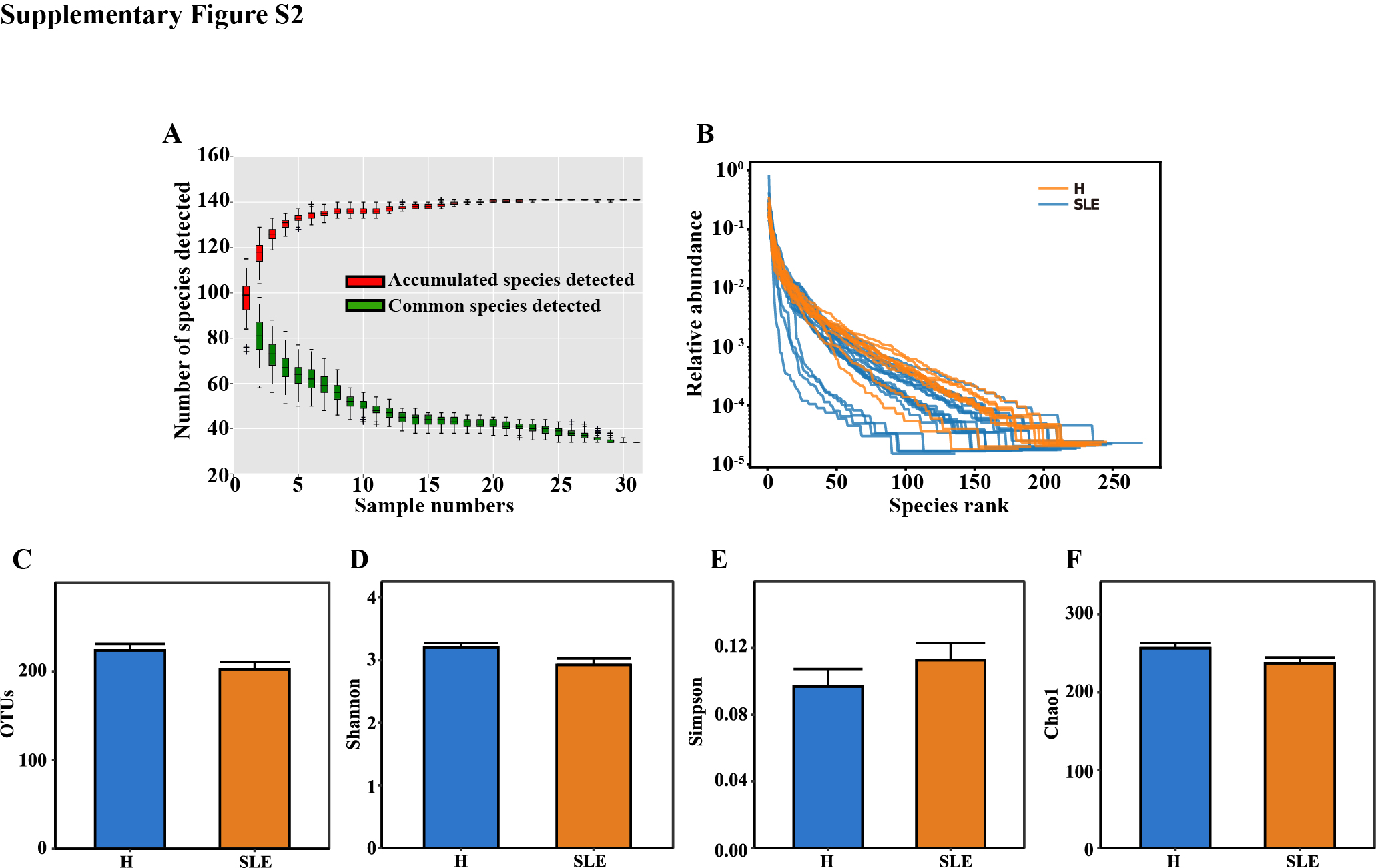

Supplement: Supplementary file 2 [file Image_2.JPEG]

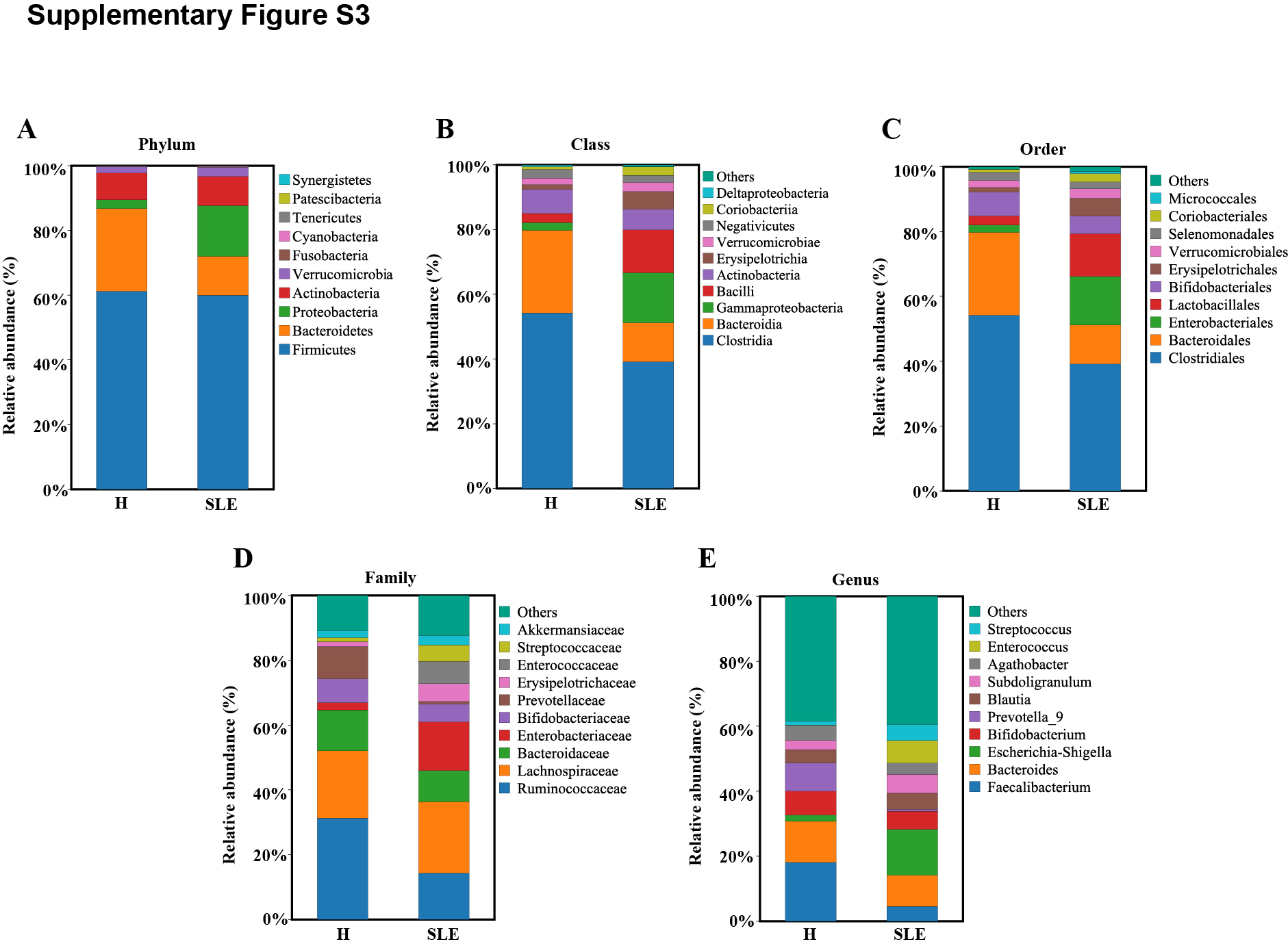

Supplement: Supplementary file 3 [file Image_3.JPEG]

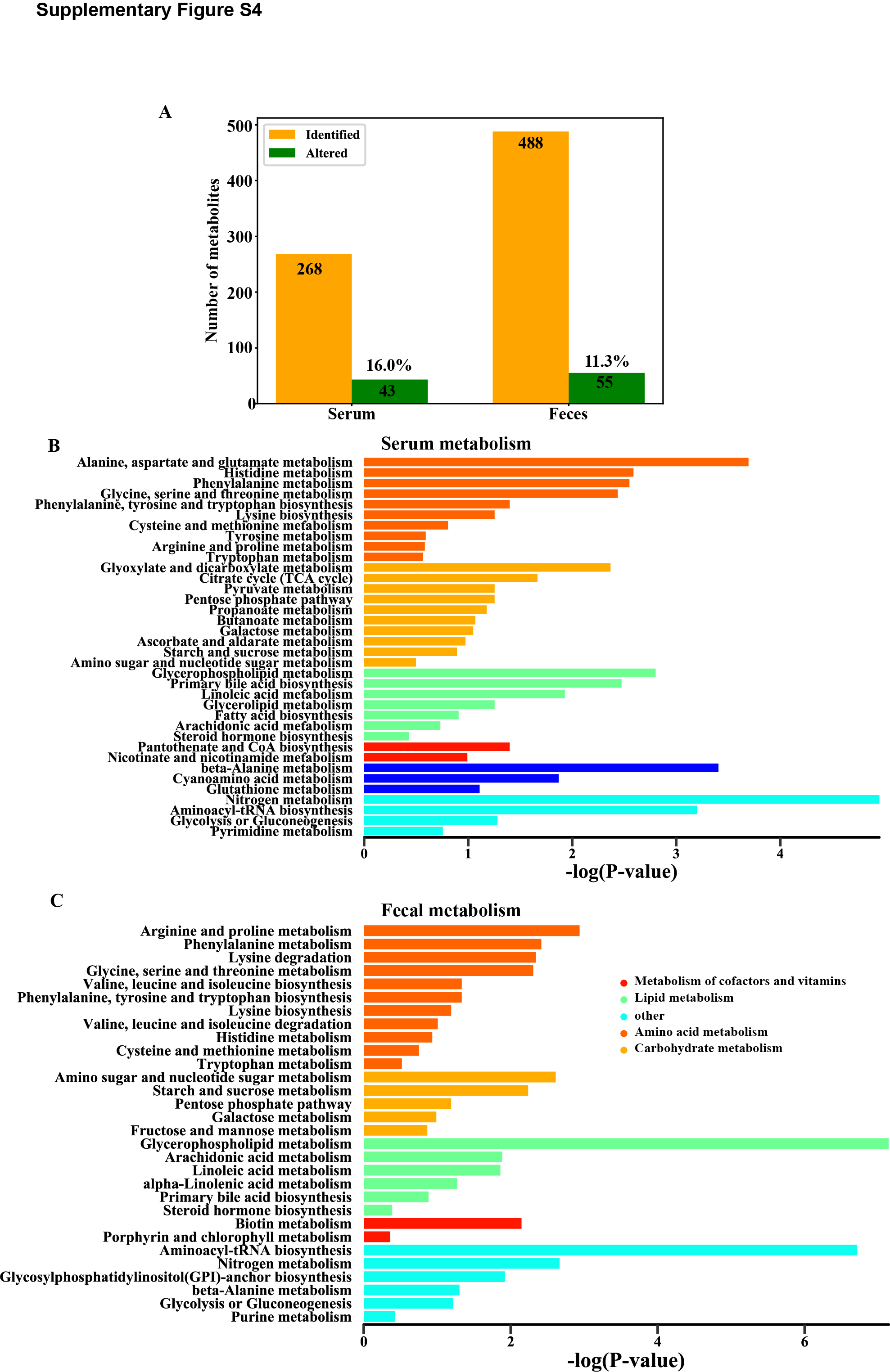

Supplement: Supplementary file 4 [file Image_4.JPEG]

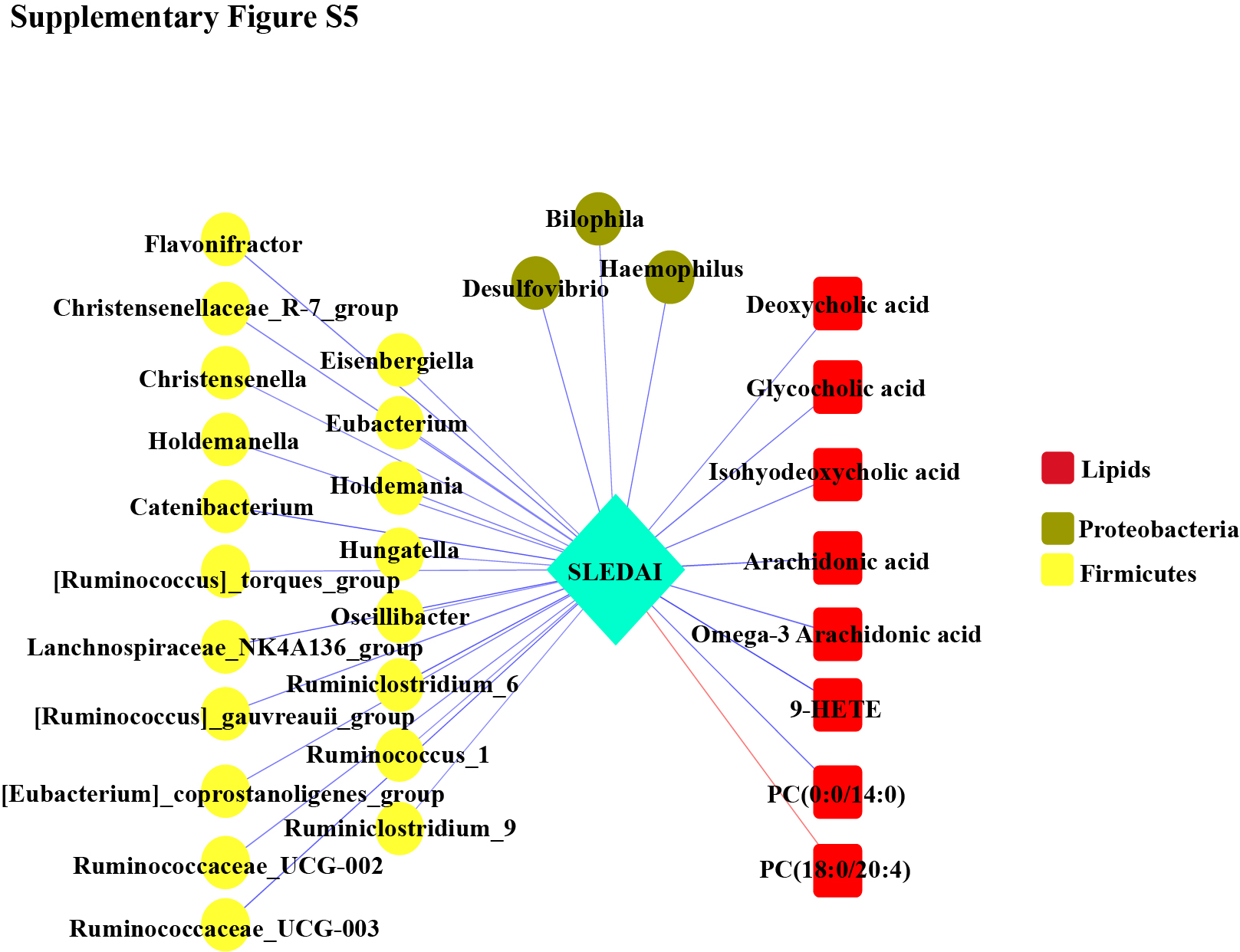

Supplement: Supplementary file 5 [file Image_5.JPEG]
